# Molecular Dynamics Simulation Studies of GLUT4: Substrate-Free and Substrate-Induced Dynamics and ATP-Mediated Glucose Transport Inhibition

**DOI:** 10.1371/journal.pone.0014217

**Published:** 2010-12-03

**Authors:** Suma Mohan, Aswathy Sheena, Ninu Poulose, Gopalakrishnapillai Anilkumar

**Affiliations:** Amrita School of Biotechnology, Amrita Vishwa Vidyapeetham, Kollam, Kerala, India; City of Hope National Medical Center, United States of America

## Abstract

**Background:**

Glucose transporter 4 (GLUT4) is an insulin facilitated glucose transporter that plays an important role in maintaining blood glucose homeostasis. GLUT4 is sequestered into intracellular vesicles in unstimulated cells and translocated to the plasma membrane by various stimuli. Understanding the structural details of GLUT4 will provide insights into the mechanism of glucose transport and its regulation. To date, a crystal structure for GLUT4 is not available. However, earlier work from our laboratory proposed a well validated homology model for GLUT4 based on the experimental data available on GLUT1 and the crystal structure data obtained from the glycerol 3-phosphate transporter.

**Methodology/Principal Findings:**

In the present study, the dynamic behavior of GLUT4 in a membrane environment was analyzed using three forms of GLUT4 (apo, substrate and ATP-substrate bound states). Apo form simulation analysis revealed an extracellular open conformation of GLUT4 in the membrane favoring easy exofacial binding of substrate. Simulation studies with the substrate bound form proposed a stable state of GLUT4 with glucose, which can be a substrate-occluded state of the transporter. Principal component analysis suggested a clockwise movement for the domains in the apo form, whereas ATP substrate-bound form induced an anti-clockwise rotation. Simulation studies suggested distinct conformational changes for the GLUT4 domains in the ATP substrate-bound form and favor a constricted behavior for the transport channel. Various inter-domain hydrogen bonds and switching of a salt-bridge network from E345-R350-E409 to E345-R169-E409 contributed to this ATP-mediated channel constriction favoring substrate occlusion and prevention of its release into cytoplasm. These data are consistent with the biochemical studies, suggesting an inhibitory role for ATP in GLUT-mediated glucose transport.

**Conclusions/Significance:**

In the absence of a crystal structure for any glucose transporter, this study provides mechanistic details of the conformational changes in GLUT4 induced by substrate and its regulator.

## Introduction

The transport of glucose across the biological membrane is mediated by facilitative transporters called glucose transporters (GLUTs), which belong to the major facilitator superfamily (MFS), the largest family of secondary active transporters that utilize the solute gradient for the transport of substrates [1]. Based on the substrate specificity, GLUT family is divided into 3 classes [2]. GLUT4 is a member of Class I subfamily and transports D-glucose across the membrane. It is mainly expressed in adipocytes and muscle cells, and plays a major role in insulin-mediated blood glucose homeostasis [3]. In the absence of insulin, the majority of GLUT4 expressed is confined to intracellular compartments. Insulin binding to the insulin receptor activates downstream signaling that leads to the translocation of GLUT4 from the intracellular compartments to the plasma membrane where it performs glucose transport [4]–[6]. The complete translocation of GLUT4 to the membrane in response to insulin requires the co-operative action of PI3-kinase and TC10 pathways [7]. Any defect in GLUT4 trafficking pathway may lead to insulin resistance, a hallmark of type 2 diabetes. Several proteins involved in the signal transduction pathways governing GLUT4 trafficking have been suggested to have therapeutic potential [8].

Insulin-stimulated glucose transport requires not only the translocation of GLUT4 to the plasma membrane but also a second step known as intrinsic transporter activation at the plasma membrane [9]. Though the exact mechanism involved in this activation process is unknown, biochemical studies suggest an inhibitory role of phosphorylation on GLUT4 intrinsic activity [10], [11]. The major site of GLUT4 phosphorylation has been mapped to Ser488, which lies in the carboxy terminal region of the molecule [12]. The intrinsic transporter activation of GLUT4 is also regulated by protein-protein interactions. Binding of hexokinase II to GLUT4 was found to be inhibitory to this transport activation, and this inhibition could possibly be relieved by the binding of glyceraldehyde 3-phosphate dehydrogenase (GADPH) at the cytoplasmic terminus of GLUT4 [13]. In addition to protein binding, small molecules such as genistein [14], myricetin [15] have also been implicated in the regulation of GLUT4 transport activity. In the case of erythrocyte glucose transporter GLUT1, a direct binding of ATP has been shown to suppress its intrinsic catalytic activity [16]. An inhibitory role for ATP was reported for insulin-stimulated glucose transport in fat cells [17], suggesting that ATP may play a similar role in regulating GLUT4 activity as well.

GLUT4 comprises 509 amino acids with 12 helices traversing through the lipid bilayer, and a large cytoplasmic loop located between transmembrane helices 6 and 7 [18], [19]. Both N and C termini are cytoplasmic, and these end regions together with the loop regions possess the distinct transporter specific signature sequences involved in the spatial regulation of GLUT4 [20], [21]. A glycosylation site is present in the extracellular loop connecting TM1 and TM2 [22]. Very little biochemical and biophysical studies have been conducted towards understanding the structure and function of GLUT4. However, a wide range of studies are available on GLUT1, a close homolog of GLUT4. These studies revealed that transmembrane segments 1, 2, 4, 5, 7, 8, 10 and 11 form the glucose transport channel and its amphipathic nature suggests the possibility of an aqueous permeation pore for the transport of glucose [23]–[30]. The outer helices 3, 6, 9 and 12 stabilize the central channel [31]–[34]. Several amino acid residues important for the function of glucose transporters have been identified from mutagenesis studies, and many of these residues are conserved among the GLUT members. Noticeably, ATP is shown to regulate the GLUT1-mediated glucose transport activity, but it does not require any ATP hydrolysis [35]–[37]. Further studies revealed that ATP binds to the Walker B motif located at the cytoplasmic loop between TM8 and TM9 [38]. Our studies with GLUT4 identified a similar Walker B nucleotide binding motif between TM8 and TM9, thereby suggesting a possibility of ATP-regulated glucose transport by GLUT4 [39].

**Figure 1:**
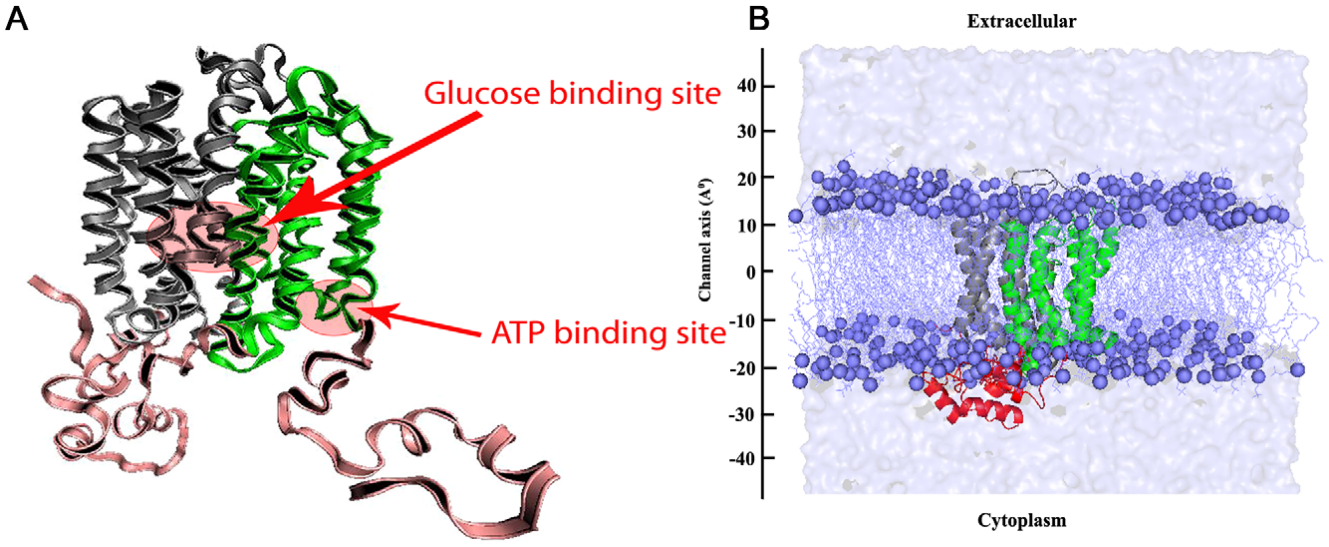
Homology model of GLUT4 and the simulation system. (**A**) A cartoon representation of the GLUT4 homology model used for simulation studies with the two half helix bundle domains D1 and D2 colored in grey and green respectively. The N- and C-terminal end regions and the long cytoplasmic loop regions are colored in pink. Glucose and ATP binding sites also marked. (**B**) Snapshot of a representative simulation system. The protein is shown in cartoon representation. The P atoms of POPC lipid bilayer are shown in spheres and the rest of the bilayer as lines. The light blue color surface representation shows the water box.

There are no crystal structures available for any members of the facilitated glucose transporter family. In this context, homology modeling approaches have been used to elucidate the structural details of GLUT members [15], [39]–[44]. The crystal structure information available for the bacterial MFS members has provided a framework for the homology modeling studies. We recently proposed a homology model for GLUT4 based on glycerol-3-phosphate transporter (GlpT) from *E. coli* (Figure 1A) [39]. This model has been validated with known substrates and inhibitors and used to explain the inhibition mechanism for GLUT4-mediated glucose transport by kaempferitrin [45]. Homology modeling and subsequent molecular dynamics simulation studies have been used to understand the structure and dynamic behavior of many membrane proteins. Such studies have provided significant insights into the structure-function relationship of many membrane proteins like GABA receptors [46]–[49], dopamine receptor [50], ion channels [51]–[54] and GPCRs [55]–[57].

In the present study, we have used a well validated GLUT4 homology model for the molecular dynamic simulations in a lipid bilayer environment to gain an insight into the intrinsic dynamic behavior, substrate-induced conformational changes and the role of ATP in the regulation of GLUT4-mediated glucose transport. Separate simulations were conducted to investigate the conformational behavior of the transporter in three different states namely; substrate free (apo), glucose bound and glucose-ATP bound forms. The latter two forms were obtained by docking studies. Our studies demonstrate significant domain rearrangements in the two half helix bundles of GLUT4 among the three different simulations. These distinct rearrangements in the transporter are mediated by an interesting pattern of inter-helical hydrogen bonds and salt bridges. Interestingly, the simulation studies with ATP suggest a potential role for ATP in modulating GLUT4 transport activity.

## Results and Discussion

We performed molecular dynamics simulation studies of GLUT4 embedded in the POPC (1-palmitoyl 2-oleoyl-sn-glycero-3-phosphatidylcholine) lipid bilayer environment to investigate 1) the intrinsic dynamic behavior, 2) the substrate-mediated conformational changes, and 3) the role of ATP in regulating the activity of GLUT4. The initial structure used for these simulation studies was from the previously established homology model of GLUT4 [36]. The model we generated has an inward facing orientation and is in agreement with the crystal structure of GlpT, the template used for modeling. Simulations were carried out with the starting structures corresponding to GLUT4-apo, glucose bound and glucose-ATP bound forms. The ligand bound forms were obtained from the docking studies. Glucose was docked to the glucose transportation pore and ATP was docked near the ATP binding motif located at the loop between TM8 and TM9 (Figure 1A). The simulation system is shown in Figure 1B and the simulation details are summarized in Table 1.

**Table 1:** Overview of the MD simulations performed.

Run ID	Duration	Description	Components
MD0	20 ns	Apo-GLUT4 protein.	GLUT4
MD1	20 ns	Glucose bound to glucose transportation channel of GLUT4.	GLUT4+Glucose
MD2	20 ns	Glucose bound to glucose transportation channel and ATP bound to ATP binding site of GLUT4.	GLUT4+Glucose+ATP

### Conformational Stability and Flexibility

**Figure 2:**
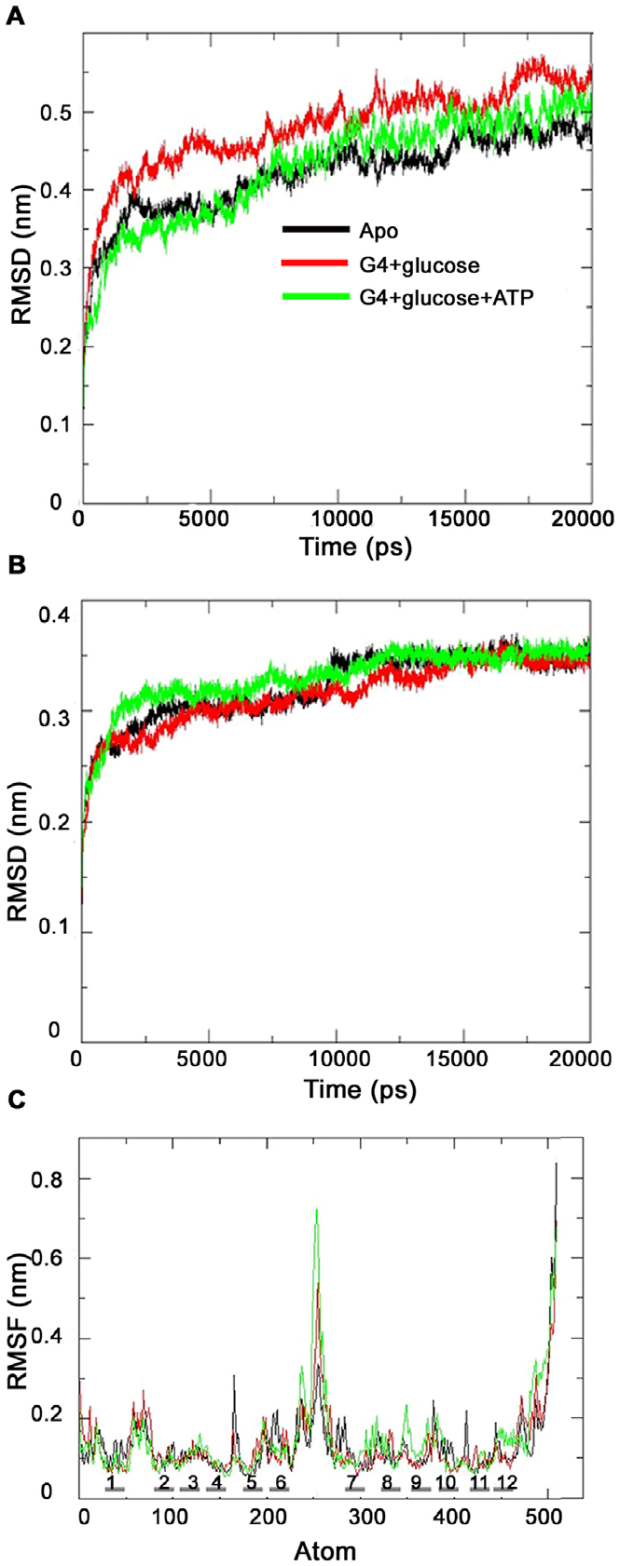
RMSD (Root Mean Square Deviation) and RMSF (Root Mean Square Fluctuation) profile. (**A**) The Cα RMSD calculated for the 3 simulation systems. (**B**) RMSD analysis for the region excluding N-terminal, C-terminal ends and cytoplasmic loop between TM6 and TM7. (**C**) RMSF of each Cα atom as a function of time. The 12 transmembrane regions are also marked. In the figure apo, glucose and glucose-ATP bound forms are shown in black, red and green respectively.

The Root Mean Square Deviation (RMSD) of Cα atoms with respect to the initial conformation was calculated as a function of time to assess the conformational stability of the protein during the simulations (Figure 2A). An initial steep rise in the RMSD for the first ∼2.5 ns and subsequently a slow increase was observed for the three simulation systems MD0, MD1 and MD2 with a final RMSD of 4.7 Å, 5.3 Å, 5.0 Å respectively (Table 2). The long N terminus, C terminus and the cytoplasmic loop between TM6 and TM7 showed significant fluctuations. When these regions were excluded from the model, the three systems exhibited comparatively lesser deviation and stabilized around 10 ns (Figure 2B). This result implies the compactness of the fold of GLUT4 during the simulation time scale. In order to analyze the inter- and intra-domain fluctuations that could eventually mediate the overall rearrangement of the transporter, the RMSD of the D1 domain (TM1-TM6) and D2 domain (TM7-TM12) were analyzed. In our simulation studies, domain D1 has shown the major conformational changes both in the case of GLUT4-apo and glucose bound forms (Table 2). Similar observations were obtained in the simulation studies with ligand bound form of one of the well characterized MFS family members, LacY [58]. NMR studies have also shown that LacY domain D2 secondary structure is more stable than that of domain D1 [59]. In our glucose-ATP bound form simulation, domain D2 exhibited a significant conformational rearrangement and is seen as elevated RMSD. This indicates that domain D2 may have a role in the regulation of ATP-mediated glucose transport (Table 2).

**Table 2:** RMSD of three simulations.

Simulation	Components	RMSD (Å)
MD0	All Cα	4.7
	CORE	3.5
	D1	2.8
	D2	2.5
MD1	All Cα	5.3
	CORE	3.5
	D1	2.8
	D2	2.6
MD2	All Cα	5.0
	CORE	3.5
	D1	2.3
	D2	3.0

RMSD of the Cα atoms with respect to the starting structure (average of the last 5 ns simulation). CORE represents the Cα atoms of GLUT4 except the long N-terminus, C- terminus and the cytoplasmic loop between TM6 and TM7.

To identify the flexible regions of the protein, Root Mean Square Fluctuation (RMSF) of Cα atoms from its time averaged position was analyzed (Figure 2C). The loop regions showed large fluctuations irrespective of the simulation system. Lower flexibility was observed for the TM segments, and this was in agreement with the stability of the helical bundle region observed from the RMSD analysis. Relatively high RMSF values were observed for the C-terminal half bundle domain in the glucose-ATP bound form compared to the other two forms. This indicates an ATP-mediated conformational rearrangement in the C terminal domain, and is consistent with the observation from RMSD analysis.

### Principal Component Analysis (PCA)

**Figure 3:**
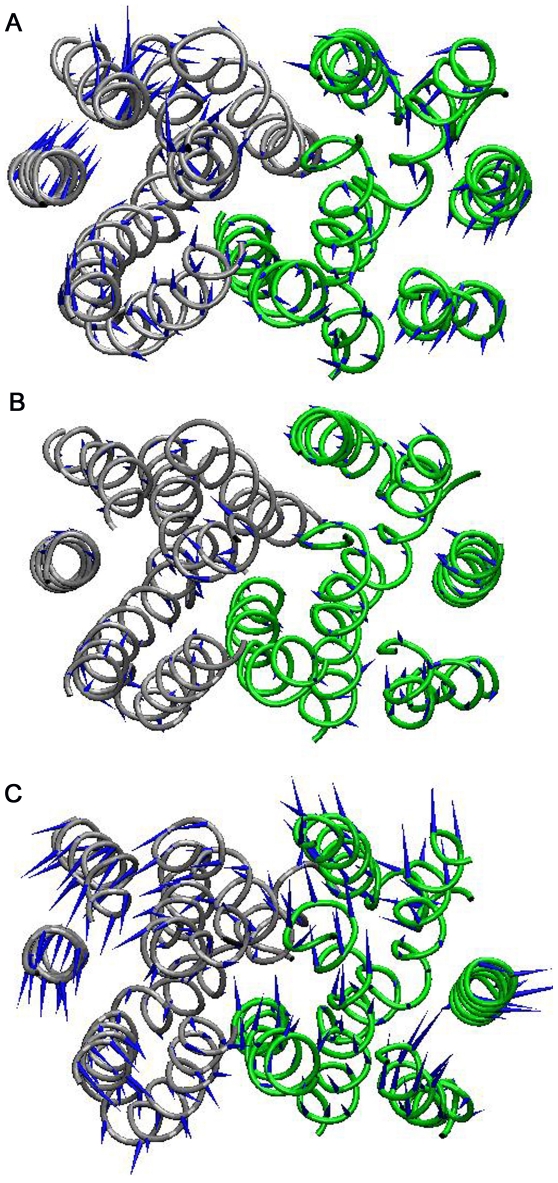
Dominant motions in the three simulation systems using Principal Component Analysis (PCA). (**A**) Porcupine plot of the first eigen vector from the apo (**B**) glucose bound (**C**) glucose-ATP bound simulations. For clarity, loop regions are not shown in the figure.

The prominent motions in the transporter during the course of simulation were analyzed with the help of PCA [60]. The principal motions for first eigen vectors of the three simulations are visualized using porcupine plot (Figure 3). First eigen vector account for 37%, 42% and 38% of the motions in apo, glucose and glucose-ATP bound simulations, respectively. We observed a concerted movement of TM helices within domains D1 and D2 during the simulations, suggesting a rigid body movement of these domains. The noticeable motion in the apo form simulation was a clockwise rotation of the two domains relative to one another, and is shown as a schematic diagram in Figure 3A. While in the case of glucose-ATP bound form, domains showed an anti-clockwise rotation type movement, except TM9 in domain D2 which showed a different direction of motion (Figure 3C). However, the molecular dynamics of glucose bound form of the transporter lacked any recognizable movement probably suggesting a more stable conformation for the transporter in the substrate bound state (Figure 3B). Hydrogen exchange kinetic studies with purified GLUT1 in the presence or absence of glucose also showed a more stabilized protein structure in the substrate bound form supporting our observation made from simulation studies [61].

### Transport Channel Analysis

**Figure 4:**
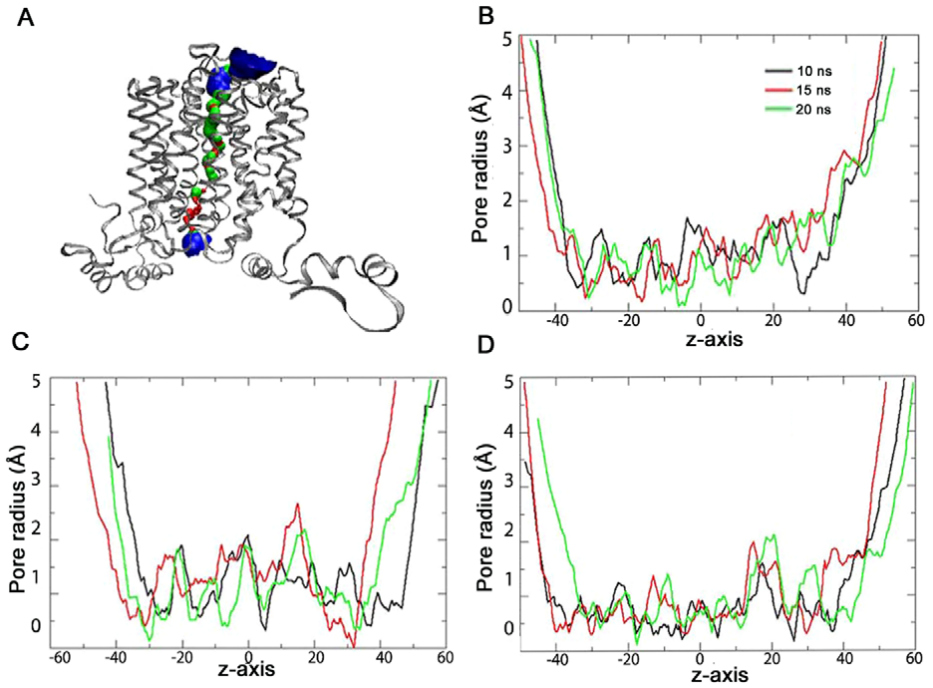
Pore radius profile. (**A**) The glucose transport channel at the end of apo form simulation. Pore radius below 1.15 Å in red, between 1.15 Å and 2.30 Å in green and above 2.30 Å in blue color representation. Pore radius profile for the (**B**) apo, (**C**) glucose bound and (**D**) glucose-ATP bound forms at different intervals.

The channel pore was monitored during the simulations using the HOLE program. We observed profound differences in the channel during the three simulations. The transporter undergoes significant conformations depending on the simulation system. Initially, our GLUT4 model was in a conformation opened to the cytoplasmic side of the transporter [39]. In the apo form, the cytoplasmic portion of the channel showed a tendency towards a closed conformation, whereas the extracellular region displayed an open state conformation (Figure 4A and 4B). The apo form simulations of other MFS members like LacY [58] and GlpT [62] have also reported a similar conformation. The observed events in the apo form simulation (i.e., the cytoplasmic end closing and extracellular end opening) may facilitate the transporter to rearrange its conformation so that the extracellular substrate binding site will be accessible for the substrate. These results indicate a rocker switch type of domain movement proposed for the MFS transporters [63]. In the presence of glucose and glucose-ATP, the channel showed a constricting type of behavior mainly at both ends (Figure 4C and 4D). This may be an occluded conformation when the substrate binds to the transporter. It is important to note that the experimental structure of two MFS transporters, EmrD and OxlT also exhibited an occluded state conformation in the presence of substrate [64], [65].

### Inter-helical Hydrogen Bonds

Hydrogen bonds formed between TM helices are shown to play a critical role in stabilizing the tertiary structure of membrane proteins as well as in the conformational rearrangement required for specific functions [66]. We analyzed all possible inter-helical hydrogen bonds formed by GLUT4 during the time period of molecular dynamics simulation. Several inter and intra-domain hydrogen bonds were identified, and significant rearrangements of the hydrogen bonds were also observed in the three simulation systems. The centrally located TM segments, TM1 and TM7 interact with most of the other helices through a number of hydrogen bonds. We observed some stable hydrogen bonds throughout the simulations (Table 3).

**Table 3:** Inter-helical hydrogen bonds.

TM	MD0	MD1	MD2
TM1-TM3	Y40-N116Q37-N116	Y40-N116	Y40-N116N116-Q37
TM1-TM4	N41-S149//N41-S153Y40-T152S30-Y159	N41-S149	N41-S149//N41-G146Y40-S149S30-Y159
TM1-TM5	S35-N176	A179-S35	
TM1-TM6	S30-F222	Q216-Q37	Q37-Q216
TM1-TM7	K50-Y309	K50-Y309	K50-Y309
TM1-TM11	W428-N41		N46-N431
TM2-TM4	S95-A147	S95-A147	S95-A147
TM2-TM11	S89-T429S89-N431	S89-T429S89-F432//N431-S89	
TM3-TM4			N115-Y148T152-N115//T152-N116
TM3-TM6	N116-Q216	N116-A213N116-Q216Q216-M112	S124-L205S124-T209
TM5-TM6			L208-L190
TM5-TM7	Q188-A305Q177-S297		Q188-A305
TM5-TM8		Q177-T337	
TM5-TM10	N176-W404		Q177-W404
TM5-TM11		N176-G424	Q177-N427N176-A421
TM7-TM8			N304-N333
TM7-TM10	A305-E396S301-E396S297-G400	A305-E396N304-E396S301-E396S297-P399	N304-F389S301-E396
TM7-TM11	Q299-S430Q298-N427I434-Q299N427-L294S426-Q295	Q295-S430	I434-Q299S430-Q298T429-Q295
TM8-TM10	N333-E396	N333-A393	N333-E396

Stable hydrogen bonds having more than 50% occupancy. //represents bonds formed alternatively.

Several hydrogen bonds were observed within domains D1 and D2. The stable nature of the hydrogen bond network within these domains suggested the possibility of an intra-domain helical packing. Among the amino acids involved in the formation of stable intra-domain hydrogen bond, mutagenesis studies on the following residues showed a reduced rate of glucose transport: S35(S23), Q37(Q25), M112(M96), Y159(Y143), Q216(Q200) [24], [29], [32], [34] in domain D1, and T326(T310), N333(N317), E396(E380), G400(G384), P399(P385), N427(N411), W412(W428), N431(N415) in domain D2 [25], [27], [28]. The corresponding residues in GLUT1 are included in the parentheses. Since our simulation studies showed an importance for these residues in the formation of hydrogen bond network, it is possible that these residues may aid in orienting the transmembrane helices in the helix bundle by interacting with neighboring helices. This might help in the stabilization of GLUT4 conformation. Such inter-helical hydrogen bonds were shown to stabilize the tertiary structure of membrane proteins, and the rearrangements of these bonds were required for conformational flexibility. For example, it has been shown that the inter-helical hydrogen bonds contribute to the stabilization of protein tertiary structure in GPCR [67]. Similarly, the active conformation of the thyrotropin receptor is attained by the release of an inter-helical hydrogen bond [68]. Furthermore, mutation of a single polar residue in the cystic fibrosis transmembrane conductance regulator (CFTR) protein resulted in the formation of hydrogen bond causing a conformational change leading to loss of protein function [69], [70].

**Figure 5:**
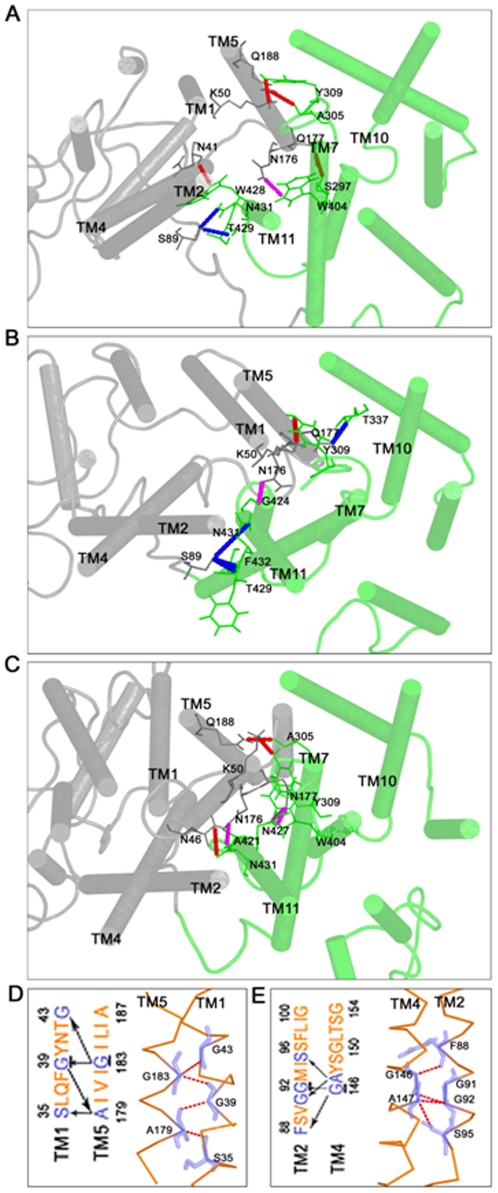
Inter-helical hydrogen bonds formed during the three simulations. The extracellular view of the stable inter-helical hydrogen bonds observed in the (**A**) apo (**B**) glucose-bound (**C**) glucose-ATP bound forms. The transporter is shown as cylindrical cartoon with domain D1 in grey and domain D2 in green. Hydrogen bond forming residues are represented with lines and hydrogen bonds are highlighted with dashed lines. The hydrogen bonds at the TM5-TM8 and TM2-TM11 interface (blue), the cytoplasmic half (magenta), and the extracellular half (red) are shown. (**D**) Residues at the TM1-TM5 interface and interactions at the TM1-TM5 interface. (**E**) Residues at the TM2-TM4 interface and interactions at the TM2-TM4 interface. The highly conserved residues across the GLUT family, G39 in TM1, G183 in TM5, G92 in TM2 and G146 in TM4 are underlined.

Small residues such as glycines are known to mediate helix packing in polytopic membrane proteins [71]. This is mainly by the presence of GxxxG motif that favors the interactions between helices mediated by Cα-H…O hydrogen bonds [72], [73]. Small residues like alanine, valine or serine could be substituted for glycine. This motif is known to mediate oligomerization in glycophorin A [74]. GxxxG motif has also been shown to mediate helix-helix interactions in various proteins like glycerol facilitator (GlpF) [75], calcium ATPase [73] and major intrinsic proteins (MIPs) [76]. In GLUT4, we observed GxxxG motif at the TM helices, 1, 2, 3, 4 and 5 in domain D1 and none in domain D2. Further analysis suggested that these motifs were located at the interfaces between TM1-TM5 and TM2-TM4 (Figure 5D, and 5E). Our simulation studies have shown the Cα-H…O hydrogen bonds between these TM helices using a Cα-acceptor distance cut-off 3.5 Å (Figure 4D and 4E arrows). Exhaustive mutagenesis studies with the highly conserved residues in the TM1-TM5 and TM2-TM4 interface G183(G167 in GLUT1), G39(G27), G92(G76), G146(G130), and the TM1-TM5 interface, S35 (S23), A187 (A171), F88 (F72), G91 (G75), G92 (G76), S96 (S80), A147 (V131) have demonstrated their role in glucose transport activity [24], [26], [29], [30]. The residues S35(S23), G39(G27), G183(V167), A187(A171) in TM1-TM5 interface, and G146(G130), G150(G134) in TM2-TM4 interface are anchor points in the GxxxG motif. Mutations in these residues resulted in lower levels of GLUT4 protein associated with the plasma membrane. One probable reason for the reduced surface expression could be that these mutants had dominant structural rearrangements leading to misfolding in the endoplasmic reticulum and chaperone-mediated protein degradation. It is also possible that the conformational changes in the protein prevented the binding of key regulators of protein trafficking and docking of GLUT4 on to the plasma membrane. These findings highlight the significant role of GxxxG motif in the helical packing of TM5 with TM1, and TM4 with TM2 as well as in maintaining the proper conformation of GLUT4. The importance of the helix packing between TM1-TM5 and TM2-TM4 for the substrate transport process was suggested in studies with LacY [77].

A domain rearrangement during the substrate transport process was observed in our simulation studies similar to one reported between the two domains of MFS transporters [63]. This suggested the importance of analyzing the inter-domain interactions. We observed several stable hydrogen bonds and salt bridges playing a role in this process. In GLUT4, the hydrogen bonds were formed mainly at the TM1-TM7, TM1-TM11, TM2-TM11, TM4-TM11, TM5-TM7, TM5-TM8, TM5-TM10, and TM5-TM11 interfaces. The main contact face between the two domains was at the TM2-TM11 and TM5-TM8 interfaces. In the apo form, stable interactions were found at the TM2-TM11 interface and weak interactions were observed at the TM5-TM8 interface (Figure 5A). Stable hydrogen bonds were seen at both contact interfaces in the glucose bound form (Figure 5B). In addition to all the stable hydrogen bonds formed at these interfaces in the apo form, one more stable interaction was observed at the interface TM2-TM11 in substrate bound form. This additional interaction may contribute towards the generation of an occluded conformation for glucose bound GLUT4. The apo form simulations of MFS protein LacY have revealed an inward displacement of transmembrane helices TM4, TM5, TM10 and TM11. However the importance of hydrogen bonds for such helical displacement was not discussed [58]. In this study, TM4 also showed similar displacement like LacY, but there was no stable inter-domain hydrogen bond formed by that TM segment. The interactions at these helical interfaces TM2-TM11 and TM5-TM8 were disrupted in glucose-ATP bound form due to the rearrangements of TM5 and TM11 (Figure 5C). It is interesting to note that three simulations have shown a channel closing type of movement at the cytoplasmic end with a more pronounced effect for the glucose-ATP bound form of GLUT4. It can be explained by the fact that one could see three strong hydrogen bonds in the glucose-ATP bound form among TM5, TM10 and TM11 (Figure 5C). But the stable interaction was observed in the apo form between TM5 and TM10, whereas in glucose bound form, it was seen between TM5 and TM11. These inter-domain hydrogen bonds in the glucose-ATP bound form enable the transporter to attain a more compact interface between the domains at this end. This observation was further supported by our pore radius profile and salt bridge analysis. At extracellular half of the channel, the inter-domain hydrogen bonds appear to line the channel in the apo form (Figure 5A). Among those, the hydrogen bond formed by K50-Y309, between TM1 and TM7 was found to be stable in all the simulations. This information is consistent with the mutagenesis studies on residue Y309, suggesting a significant role for this residue in channel closing at the extracellular end [78]. In the glucose bound form, all other hydrogen bonds at the extracellular half were weak, (Figure 5B) whereas prominent interactions were seen in glucose-ATP bound form (Figure 5C).

### Salt Bridge Analysis

**Figure 6:**
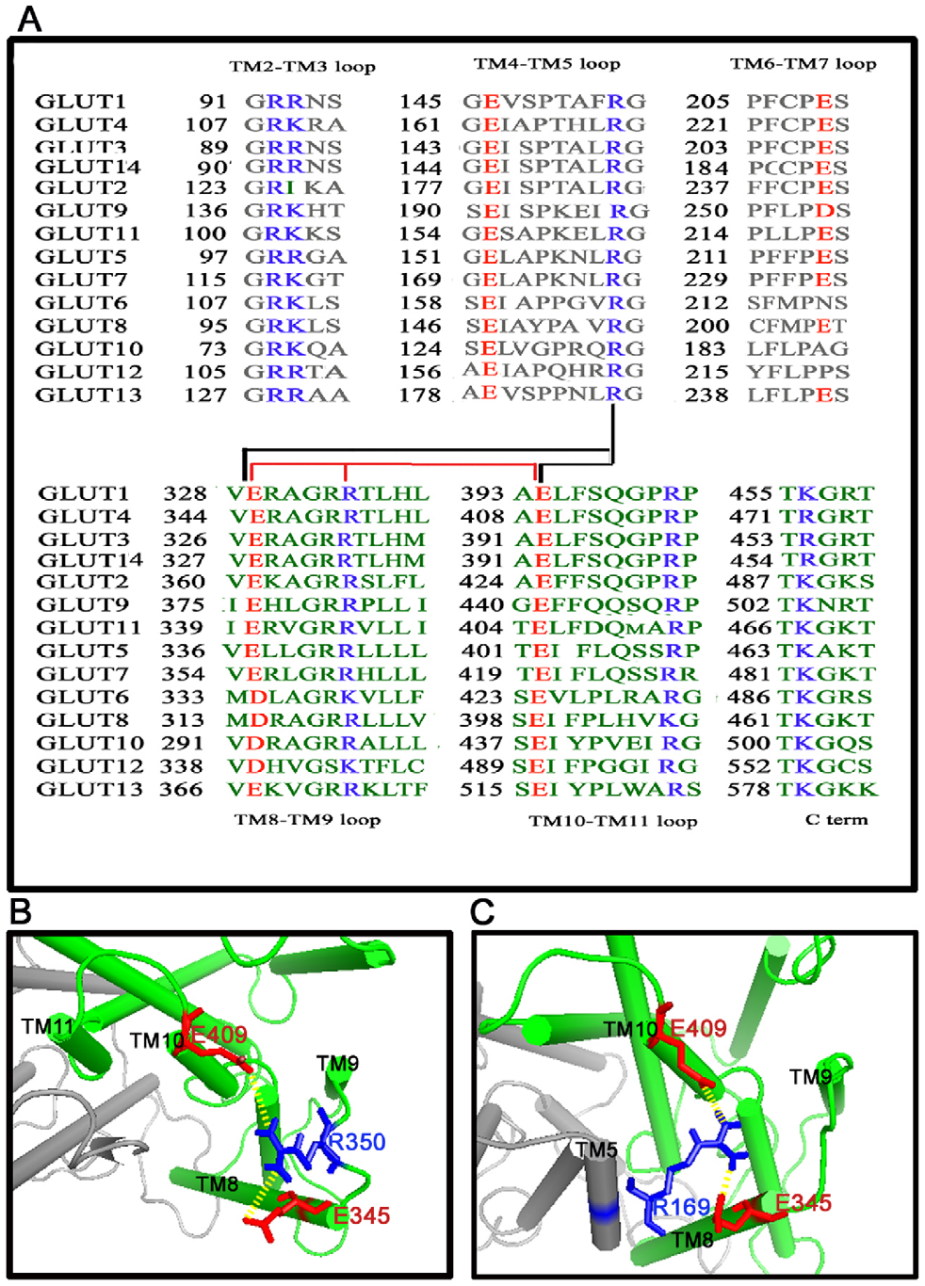
Salt bridge switching. (**A**)The sequence alignment of 14 members of GLUT family: the salt bridge forming residues are highlighted. The network of salt bridge which has a switching pattern is also highlighted. (**B**) The intra-domain salt bridge network E345-R350-E409 found with the presence of glucose. (**C**) The inter-domain salt bridge network E345-R169-E409 found with the presence of glucose-ATP.

The presence of conserved charged residues in glucose transporters has suggested the possibility of salt bridges playing a role in their conformational rearrangements. The multiple sequence alignment of all members of GLUT family revealed the conservation of acidic residues such as E162, E225, E345, E409, E470, and basic residues R108, K109, R142, R169, R228, R285, R349, R350, R416, R472 across the members. Some residues like E193, E396 and R467 showed a class I specific conserved pattern (Figure 6A). Our simulation trajectories indicate the presence of several salt bridges, and further analysis has identified an interesting pattern of salt bridge network. Both intra- and inter-domain salt bridges were observed during the simulation (Table 4).

**Table 4:** Salt bridge details.

Salt bridge	Domain	MD0	MD1	MD2
E345:R350	C	0.19–1.16 (62.5%)	**0.19–0.69 (99.2%)**	0.84–1.69
E345:R169	*Inter domain*	1.24–2.16	0.25–1.01 (12.5%)	**0.17–0.7 (99.5%)**
E409:R169	*Inter domain*	1.11–1.68	0.19–1.18 (45.7%)	**0.2–0.87 (95.1%)**
E409:R416	C	0.82–1.13	**0.18–0.83 (71.9%)**	0.24–0.87 (24.5%)
E409:R350	C	0.88–2.04	**0.18–0.91 (97.8%)**	0.47–1.18 (1.2%)
E409:R474	C	0.26–0.84 (72.0%)	**0.18–0.7 (97.4%)**	**0.2–0.85 (88.6%)**
E225:R108	N	0.44–1.07	**0.21–0.54 (100%)**	**0.31–0.63 (99.7%)**
E162:R108	N	**0.17–0.32 (100.%)**	**0.18–0.46 (100%)**	**0.18–0.39 (100%)**
E225:R110	N	**0.2–0.6 (100%)**	**0.29–0.72 (74.8%)**	**0.18–0.51 (100%)**
E225:K109	N	**0.19–0.7 (84.2%)**	**0.28–0.62 (100%)**	**0.2–0.58 (100%)**

The distance of the salt bridge formed in each simulation is given in nm. The cut-off used for this electrostatic interaction was 6 Å The distance between charged residues and percentage of occurrence of salt bridges (in parentheses) are shown. Strong salt bridges are highlighted in bold. The last 10 ns of the trajectory was used for salt bridge analysis.

Several salt bridges were observed in domain D1 and D2. Among these, the salt bridges E225:R110, E225:K109 and E162:R108 were stable in domain D1 in all the three simulation systems. Mutagenesis studies suggested that R108 located at the cytoplasmic loop between TM2 and TM3 as well as E162 at the end of TM4 play a critical role in glucose transport function [79]. But further evidence about the actual mechanism of involvement of these residues is unknown. Based on salt bridge analysis, we were able to get more insight in to the role of these residues in glucose transporter function. The salt bridge E162:R108 formed between the cytoplasmic loop of the outer helix TM3 and the channel forming helix TM4 may aid the conformational rearrangements associated with channel opening and closing. Though the residues E162 and R169 are located on the same loop, we could not observe the formation of salt bridge between these residues. In the presence of substrate, R108 forms another salt bridge with E225 located at the cytoplasmic loop between TM6 and TM7.

The salt bridges formed at the domain D2 showed drastic changes in glucose bound form. As the residues E409 and R416 are located at the same loop between TM10 and TM11 and conserved throughout the family, it implies the possibility of a salt bridge between these two residues. Our analysis has shown that this salt bridge was stable in the presence of glucose and became weak in the presence of glucose-ATP as was evident from the low occupancy value (Table 4). This may be due to the conformational rearrangements in these helices during ATP binding. The same observations were obtained for the salt bridge E409:R350 even though the residues belong to different loop regions (loop between TM10 and TM11, loop between TM8 and TM9 respectively) (Figure S1B). The salt bridge E409:R350 is pseudo-symmetric to the salt bridge E162:R108 observed in the N terminal domain and this data synchronized with the pseudo-symmetry of helix bundles. The salt bridge formed by the residues E345 and R350 was stabilized in the presence of glucose and destabilized in the presence of glucose-ATP (Figure S1A). This is mainly because in ATP bound form, E345 residue forms an inter-domain salt bridge with R169 residue located at the loop between TM5 and TM6 (Figure S1C). E409, a residue in domain D2, formed an inter-domain salt bridge with R169 in the presence of ATP (Figure S1D). Precisely there is a switching of salt bridge noticed in the presence of glucose and glucose-ATP bound forms and the intra-domain salt bridge network formed in presence of glucose E345-R350-E409 is switched to E345-R169-E409 which is an inter-domain salt bridge in glucose-ATP bound form (Figure 6A, 6B, and 6C). The interactions of ATP with the residues involved in intra domain salt bridge formation mediated the switching of the salt bridge network and thus led to subsequent conformational changes. These inter domain salt bridges and the inter domain hydrogen bonds discussed previously contribute to the ATP-mediated conformational rearrangement of the transporter. These strong interactions facilitate GLUT4 to attain a more compact conformation with a reduced pore size compared to either apo or glucose bound forms (compare Figure 4A, 4B and 4C). Moreover, these residues (R169, E345 and E409) are also shown to play a role in glucose transport activity. This is evident from the mutagenesis studies showing that mutant forms (E345, E409 and E416) are retained in the inward facing conformation [79].

The salt bridges reported in our simulations mainly reside at the cytoplasmic side of the transporter. These kinds of rearrangements of ionic interactions at the cytoplasmic end of membrane proteins are known to play a role in conformational rearrangements. For example, substrate-induced rearrangement of inter- and intra-domain salt bridges in MFS members like GlpT and LacY regulated the conformational changes of these transporters [80], [81]. In the case of GPCR, cytoplasmic salt bridges are known to play an important role in conformational transition between inactive and active states [82]–[84]. Furthermore, in OmpA ion channel, the channel opening mechanism is involved with a switching of salt bridges [85]. In the case of GLUTs, the only charged residue found in the channel is E396 located in TM10, and it does not possess a salt bridge partner. Therefore, it may be assumed that there is no salt bridge formed at the sugar transport channel in GLUTs.

### ATP Binding

**Figure 7:**
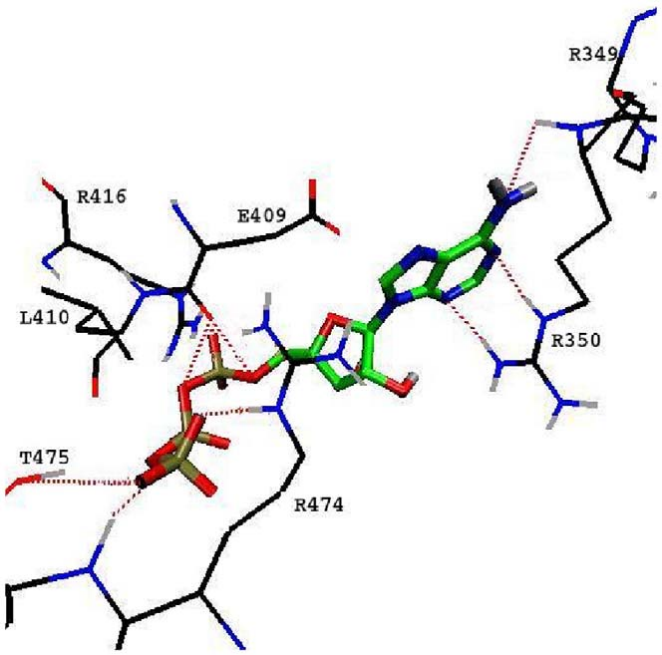
ATP interactions with GLUT4. Stable binding pose of ATP at the ATP binding site of GLUT4. The snapshot is taken from a representative of highly populated cluster of ATP bound complex obtained from the MD2 simulation trajectory.

Cytoplasmic ATP is known to regulate the glucose transport activity mediated by GLUT1, a close homologue of GLUT4 [16], [86]. In GLUT1, Walker B nucleotide binding domain located at the cytoplasmic loop between TM8 and TM9 has been shown to play a role in ATP binding [38]. Our homology modeling study with GLUT4 suggested the same Walker B motif as a potential ATP binding motif. The existence of ATP binding sites in GLUT4 was earlier proposed by Bazuine *et al*. based on inhibitor studies using genistein [14]. In the present study, ATP was docked at the ATP binding motif and the two lowest energy conformations were analyzed for the stability at the binding site. The conformation with stable binding at the active site was selected for further analysis. The residues R349, R350, E409, L410, R416, R474 and T475 are positioned around 4 Å of ATP (Figure 7). The strong interactions between ATP and the nearby residues in GLUT4 are shown in the distance plot (Figure S2). A hydrogen bond interaction between the adenosine ring of ATP and R350 was observed at the ATP binding motif. The phosphate group of ATP makes hydrogen bonds with R474, T475 residues at the C-terminal region and also with E409 at the cytoplasmic loop between TM10-TM11 of GLUT4 (Figure 7). However, biochemical studies could not assign specific roles for these residues identified in our work.

A pronounced anti-clockwise rotation of domains was observed during glucose-ATP-GLUT4 simulation. The transport channel analysis showed a narrowing of the pore radius towards the cytoplasmic end, suggesting that the transporter is in a substrate-occluded state in the presence of ATP. This observation was in agreement with a previous biochemical study in GLUT1 showing an ATP-dependent transport channel constriction [87]. The present simulation studies provide a feasible explanation for this channel closure. In comparison with the other two simulations, glucose-ATP-GLUT4 exhibited inter-domain hydrogen bonds among TM5, TM10 and TM11, making the domain interface more closely packed. Another factor contributing to this channel constriction is the switching of a salt bridge network from E345-R350-E409 to E345-R169-E409 found in the glucose-ATP bound form as described above. Mutagenesis studies in GLUT1 have suggested an important role for the residues R349 and R350 in ATP binding [38]. Levine *et al.* have proposed a proton sensitive salt bridge in GLUT1 between the conserved residues E329-R333/334 (E345-R349/350 in GLUT4) and its disruption in the presence of ATP [88]. This data lends support to our finding that the switching of salt bridge may be involved in ATP-mediated substrate occlusion in GLUT4. Based on our results, we propose a regulatory role for ATP on GLUT4 intrinsic transporter activity. It could be one way of regulating glucose uptake in response to the metabolic status in insulin sensitive cells.

### Conclusions

**Figure 8:**
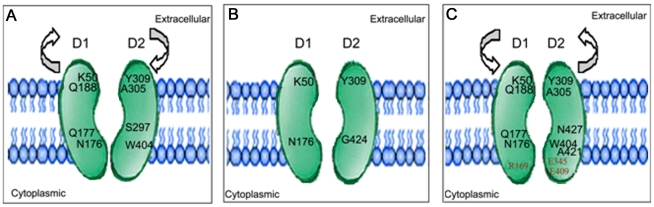
Proposed domain arrangements associated with the three simulations. (**A**) apo (**B**) glucose bound (**C**) glucose-ATP bound forms. Residues involved in inter-domain interactions are marked, inter-domain salt bridge forming residues are shown in red. The arrow head shows the direction of domain movements observed from Principal Component Analysis (PCA).

Membrane dynamics studies were used to characterize the conformational behavior of GLUT4 during different biological scenarios. The current nanosecond time-scale simulation studies provide details about the distinct conformational changes associated with different forms of GLUT4 *viz.*, apo, glucose bound and glucose-ATP bound forms. In the apo form, the transporter attains a conformation open to the extracellular region, probably via the clockwise rotation in the two domains as observed in PCA analysis (Figure 8A). This conformation may facilitate the exofacial binding of the substrate and its translocation to the cytoplasm. In the presence of substrate, the transporter attained a stable occluded conformation and this may be an immediate state before the release of substrate to the cytoplasm (Figure 8B). On the other hand, in glucose-ATP bound form, GLUT4 exhibited a more compact interface for the two domains. This conformation was achieved by the rearrangement of various inter-domain hydrogen bonds and salt bridges (Figure 8C). Thus this study explains the mechanisms related to the structural changes in GLUT4-mediated by glucose and ATP.

## Materials and Methods

### Simulation System Setup

The starting structure used for molecular dynamics simulation was the recently published homology model of GLUT4 [39]. The model is further optimized using protein preparation wizard of Schrödinger package. This optimized model has been embedded in pre-equilibrated lipid bilayer for the membrane dynamics simulation studies. The lipid bilayer system for the dynamics was built from POPC lipid bilayer, a major constituent of eukaryotic cell membrane and this was obtained from Peter Tieleman's site at University of Calgary [89]. The initial lipid bilayer obtained from the site comprised of 128 lipid molecules and to incorporate the 12 transmembrane protein, this was enlarged to a system with 512 lipid molecules. Initially, this bilayer system was equilibrated for 50 ps with position restraints on POPC and then for 7 ns without position restraints to satisfy the bilayer parameters such as area/lipid, order parmeters for the palmitoyl and oleoyl chain and electron density profile with the experimental results.

The ligand bound form was obtained by the docking of GLUT4 and ligand using the GLIDE5.0 program [90]. The glucose was docked to the QLS site and ATP was docked to the ATP binding site (Figure 1A). Among the two conformations of ATP by the docking study, one has been confirmed with a 5 ns MD simulation of GLUT4-ATP complex due to the stability of that pose in the binding site.

The prepared GLUT4 and GLUT4 ligand complexes were embedded in the pre-equilibrated lipid bilayer with the help of Visual Molecular Dynamics (VMD) [91]. The resultant system was solvated, followed by the addition of ions to neutralize the system to 0.15 M NaCl concentration. The total system was energy minimized and a 20 ps equilibration was performed on solvent and ions with position restraints on the rest of the system. The equilibration process was continued for another 4 ns with position restraints on protein for the proper positioning of lipid molecules around the protein.

### Simulation Protocol

Three separate simulations were performed on the apo, glucose bound and glucose-ATP bound forms of GLUT4. All these simulations were carried out using the GROMACS4.0.4 program [92] with gmx force field [93] at constant temperature and pressure (NPT) ensemble. The Berendsen coupling was employed to maintain a constant temperature of 310 K and constant semiisotropic pressure of 1 bar with coupling time of 0.1 ps and 1 ps respectively. A separate temperature coupling of protein, POPC and ligand, solvent and ions has been done with a coupling constant of 0.1 ps. The distance cut-off for the Coulomb and the Lennard-Jones interaction was 0.9 nm and 1 nm, respectively. The Particle-mesh Ewald method was used to treat long range electrostatic interactions. All bonds were constrained with LINCS algorithm. Time step used was 2 fs and the coordinates were saved every 10 ps for analysis. The GROMACS topologies for the ligands were obtained from PRODRG [94].

### Trajectory Analysis

Trajectories obtained from various simulations were analyzed using flexible tools provided by GROMACS. VMD was used for the visual analysis of the trajectories [91]. Wherever necessary, PyMOL was also used for the visual analysis of the snapshots [95]. Analysis of hydrogen bonds from the simulation trajectory was done with the GROMACS g_hbond utility using cut-off distance 3.5 Å and acceptor-donor-hydrogen angle 30°. In order to obtain the radius profile of the transport channel, HOLE program was used [96]. The Principal Component Analysis (PCA) was done using the Dynamite server [97]. Graphs were generated using Xmgrace [98].

## Supporting Information

Figure S1 The distance plot of the salt bridges (A) R350-E345 (B) R350-E409 (C) R169-E345 (D) R169-E409 in the three simulation systems, apo (black), glucose bound (red) and glucose-ATP bound (green) forms.
(9.50 MB TIF)

Figure S2 Distance plot of ATP binding interactions Interactions of GLUT4 residues with the (A) adenosine ring and at the (B) phosphate tail of ATP.
(8.85 MB TIF)
