# Supplementary figures and images for: Molecular Dynamics Simulation Studies of GLUT4: Substrate-Free and Substrate-Induced Dynamics and ATP-Mediated Glucose Transport Inhibition
Source: PLoS One. 2010 Dec 3;5(12):e14217. doi: 10.1371/journal.pone.0014217 (PMC2997047; doi:10.1371/journal.pone.0014217)

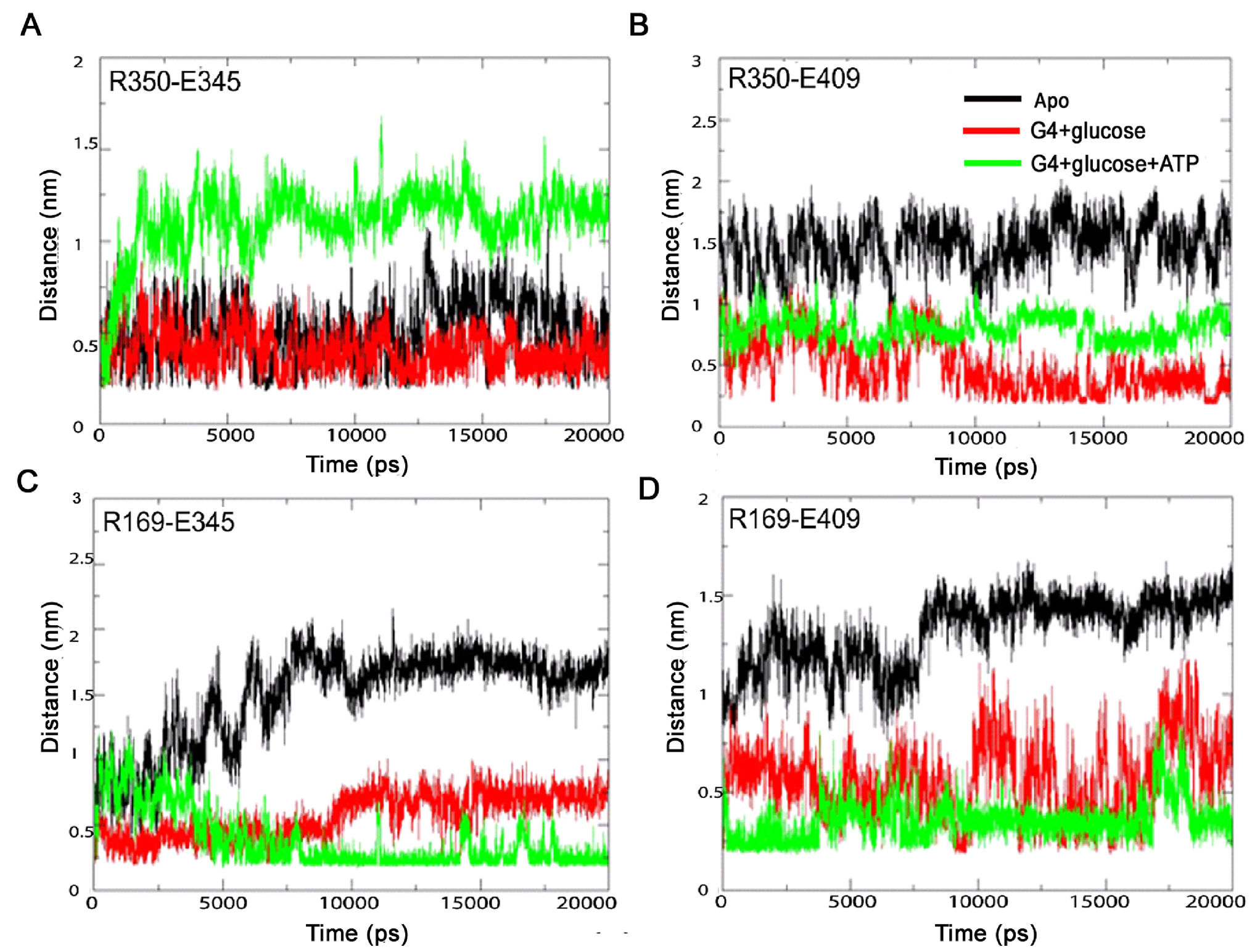

Supplement: Figure S1 — The distance plot of the salt bridges (A) R350-E345 (B) R350-E409 (C) R169-E345 (D) R169-E409 in the three simulation systems, apo (black), glucose bound (red) and glucose-ATP bound (green) forms. (9.50 MB TIF) [file pone.0014217.s001.tif]

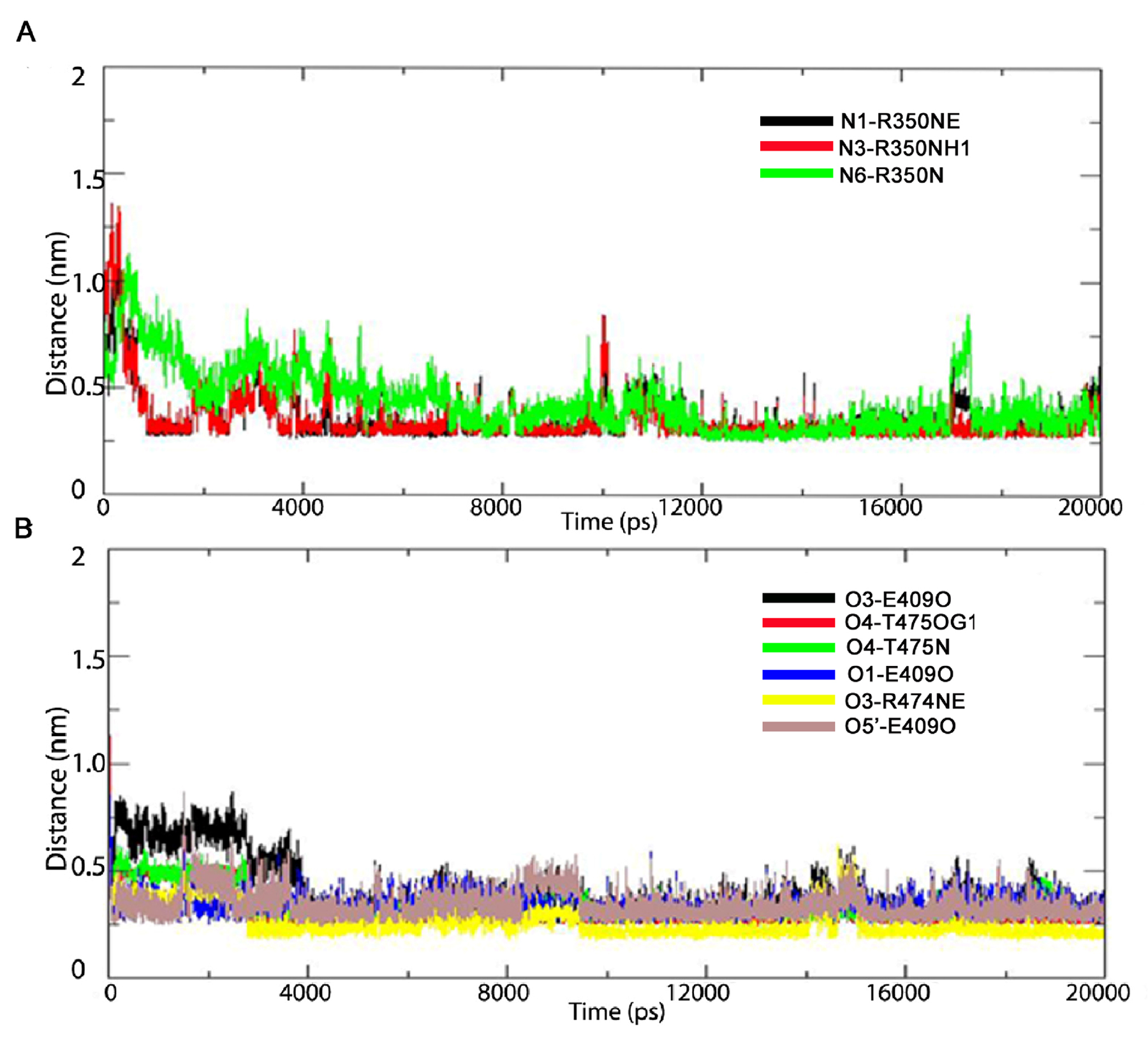

Supplement: Figure S2 — Distance plot of ATP binding interactions Interactions of GLUT4 residues with the (A) adenosine ring and at the (B) phosphate tail of ATP. (8.85 MB TIF) [file pone.0014217.s002.tif]
